# African governments must build on covid-19 responses to advance gender equality

**DOI:** 10.1136/bmj-2022-072239

**Published:** 2023-06-07

**Authors:** Kéfilath Bello, Asha George, Michelle De Jong, Oluwapelumi Adeyera, Cheikh Faye, Taiwo Oyelade, Kedest Mathews, Agnes Binagwaho

**Affiliations:** 1 Centre de Recherche en Reproduction Humaine et en Démographie, Cotonou, Benin; 2 School of Public Health, University of the Western Cape, Cape Town, South Africa; 3 Sonder Collective, Abuja, Nigeria; 4 African Population and Health Research Center, West Africa Regional Office, Dakar, Senegal; 5 World Health Organization Regional Office for Africa, Brazzaville, Congo; 6 University of Global Health Equity, Kigali, Rwanda

## Abstract

**Kéfilath Bello and colleagues** argue that building on promising practices to develop more sustainable strategies to advance gender equality will help African countries respond to future crises

Gender inequality remains a major threat to development in Africa, with millions of women in the continent not reaching their full potential.^1^ The covid-19 pandemic and related quarantine and lockdown measures exacerbated these gender inequalities. The effects included increased reports of gender based violence,^1^ ^2^ ^3^ ^4^ the economic consequences of reduced income and unemployment for women and families,^5^ ^6^ ^7^ ^8^ ^9^ ^10^ ^11^ ^12^ ^13^ and disruptions to essential health services.^14^ ^15^ ^16^ ^17^ In addition, school closures as a part of responding to the pandemic, further exposed girls and adolescents to violence, unintended pregnancies, and a risk of permanent dropout from schools across the region.^11^ ^18^ ^19^

In response, African leaders and organisations pledged to tackle the multidimensional effects of the pandemic on women and girls. For example, the African Union issued policy guidance stressing the importance of integrating gender in all covid-19 interventions.^18^ ^20^ In addition, multiple organisations working on health and socioeconomic development in the region, including civil society and UN agencies, contributed to mitigating the gendered effects of covid-19.^4^ ^21^

Three areas of intervention have been implemented widely to support women and girls during the pandemic: efforts to support female victims of gender based violence, social protection measures, and community and civil society led responses to the pandemic and associated lockdowns. Although we applaud these initial measures, they must be further strengthened to tackle the root causes of gender inequality, which are mainly structural. Structural causes are those that are beyond an individual’s direct control and refer more broadly to how economies are organised, social services are allocated, legislation made, and ideologies shaped.^22^ ^23^ We highlight opportunities to further build and strengthen initial covid-19 responses, so that they are more effective in advancing gender equality in a sustained manner across the region.

## Tackling gender based violence

In 2018, it was estimated that 33% of women and girls aged between 15 and 49 years in sub-Saharan Africa had a lifetime risk of being subjected to physical or sexual violence by a current or former partner.^24^ A rapid assessment in six Sahelian countries (Chad, Senegal, Mali, Burkina Faso, Mauritania, and Niger) showed that domestic violence, whether physical or verbal, increased from 40.6% before the covid-19 crisis to 52.2% during the pandemic in 2020.^25^ Subsequent mobile phone surveys in 2021 found that the proportion of women who reported that covid-19 had made them feel less safe at home was 45% in Kenya, 39% in Nigeria, 23% in Cameroon, 23% in Morocco, and 18% in Cote d’Ivoire.^4^ Moreover, the proportion of women who reported that physical or verbal abuse by a spouse or partner had increased in their community during the pandemic ranged from 92% in Kenya to 38% in Cameroon. 

These reports indicate the substantial scale of the problem, and the variability also reflects the challenges in collecting data on this sensitive issue. Most concerning was that women already in abusive relationships experienced heightened exposure to more frequent and severe forms of violence. Lockdown and quarantine measures meant that women were confined with their abusive partners at a time of increased stress and economic pressure while being cut off from social support and services.^2^

In 2020, 30 African countries committed to the UN secretary general’s global call to make the prevention and redress of violence against women a key part of their national response plans for covid-19.^18^ For example, the president of Liberia declared rape a national emergency in September 2020 and called for the development of a national plan across sectors to end sexual and gender based violence.^26^ The president of Kenya ordered an investigation in July 2020 into the rising reports of violence against girls and women,^27^ and in several national addresses on progress in containing the pandemic, the South African president made strong calls to fight violence against women.^18^ ^28^ ^29^

According to data from the Covid-19 Global Gender Response Tracker, the most common intervention was to establish hotlines or helplines to provide guidance and help to women experiencing gender based violence after in-person services were suspended. Helplines were implemented in 20 out of the 34 countries that reported measures against gender based violence.^30^ Governments in 18 countries in the African region, including Nigeria, Rwanda, and Somalia, also developed campaigns to raise awareness about preventing gender based violence. These included the need to involve stakeholders, particularly men, and about how victims of violence could seek help in the context of covid-19. Liberia, Tunisia, and South Africa worked on improving police and justice responses to gender based violence through offering legal aid to the victims, maintaining the gender based violence justice services during the lockdown, and strengthening the police units dealing with gender based violence. Twelve countries strengthened the accessibility of coordinated multisectoral services to women who needed care for violence. In Zimbabwe and Burkina Faso, for example, one-stop care centres were established or strengthened. Eleven countries established or kept open shelters and provided psychosocial support for women and girl survivors, while eight countries prioritised improving data collection on gender based violence.^30^

These important initiatives responded to the immediate needs of survivors of violence. But we must build on the increased political attention given to gender based violence to increase substantially the funding and scale of these measures and expand them to tackle the underlying causes of such violence. This will require focus on the structural drivers of gender inequalities through improving girls’ access to quality education and through economic and social empowerment interventions for women such as cash transfers, microfinance, and vocational training combined with communication, relationship, and life skills development.^31^

In addition, interventions directly aimed at transforming gender relations and social norms through community activism, parenting programmes, and male engagement are critical to improving gender attitudes and norms and for empowering all community members to reduce and respond to gender based violence.^31^ ^32^ Such measures would not only address the ongoing fallout from the pandemic and its social consequences but also build the foundations needed to advance gender equality as key for mitigating the negative effect of future crises on women and girls’ lives.

## Need for gender responsive and sustainable social protection systems

Although the proportion of women employed in Africa is the highest in the world, African women are more likely than women in other regions to work in the lowest paid positions^5^ ^33^ ^34^— in the informal sector and in sectors vulnerable to crisis, such as agriculture and handicrafts.^5^ ^6^ ^19^ During the first months of the pandemic, informal workers in sub-Saharan Africa, of whom 80% are women, are estimated to have lost close to 81% of their income.^5^ ^6^ ^7^ ^8^ ^13^ ^35^ There was also a larger decrease in women’s employment than men’s between 2019 and 2020 in the region,^10^ and an increase in the burden of women’s unpaid care work.^11^ ^12^ ^19^ ^35^

African governments responded to calls to mitigate the gendered socioeconomic effect of covid-19^20^ ^32^ ^36^ by introducing or expanding social protection measures for socioeconomically vulnerable households.^30^ ^37^ ^38^ Several African governments implemented cash transfers and food support programmes for informal workers (eg, Morocco, Namibia, Tunisia, Cabo Verde, Togo) 12 21 36 37 or for vulnerable women directly (eg, Egypt, Senegal, and Kenya).^39^ ^40^ In Kenya, cash transfers helped female micro-entrepreneurs during the pandemic to maintain their livelihoods, keep or re-open their businesses, and increase their business profit.^41^ In the Togolese cash transfer scheme, women were paid more than men because of the unequal care burden, and the cash transfers were shown to increase the use of healthcare services.^42^ These economic supports were not always devised as gender specific interventions, but they can have beneficial gender effects—for example, by reducing intra-household conflict, improving the emotional wellbeing and resilience of women and girls, and preventing violence against women by increasing the availability of cash to meet daily needs. Such support can improve household economic security and build women’s empowerment**.** 36 43 44 45

Despite the benefits of cash transfers programmes during the covid-19 pandemic,^38^ ^46^ concerns arose about their equitable distribution. Only 22% of the social protection measures across the African continent targeted specific gendered risks and challenges, and only 18% specifically targeted women’s economic security.^30^ Even when measures focus on women, other barriers can limit their effectiveness. While directing cash transfer payments to women through bank accounts in their name increases their control over resources,^38^ setting up an account usually requires a valid identification document or a mobile phone. However, over 45% of women in low income countries (many of which are in Africa) lacked a foundational identity document in 2018 compared with 30% of men.^47^ The digital gender divide is a major challenge in the region. In Egypt, Kenya, Nigeria, and Senegal, women were 37% less likely than men to use mobile internet in 2021, and progress in reducing this gender gap is stalling.^48^ Some policy responses to covid-19 did not adequately account for existing gender inequalities, which limited their effectiveness given that women were much more severely affected by the indirect social effects of covid-19.

**Fig 1:**
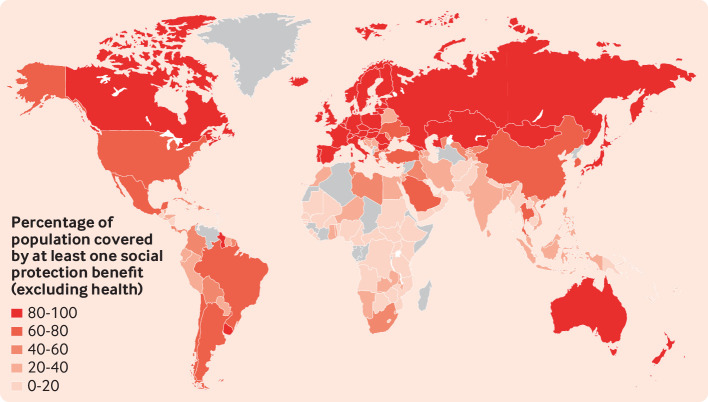
Global social protection coverage for 2020 or latest available year (data from International Labour Organization World Social Protection database)^49^

Furthermore, despite the political prioritisation of social protection during covid-19,^38^ ^46^ social protection expenditure in Africa is generally low. Most African countries spend less than 5% of their national budgets on social protection. Low income countries need to spend 15.9% of their gross domestic product (GDP) to ensure basic social protection coverage. As a result of this low investment, in many African countries less than 20% of the population is covered by at least one social protection programme (fig 1).^49^ Economic vulnerability is a key structural driver of gender inequality, and sustainable, gender sensitive economic interventions are therefore essential not only to address violence against women but to empower women and girls. Such investment is particularly critical if we are to build the social foundations to protect against future pandemics.

## Better support for communities and civil society responses

Community and civil society responses to covid-19 provided invaluable support for the health and livelihood effects experienced by vulnerable groups across the region.^3^ ^50^ ^51^ In addition to covid-19 risk communication, communities adapted approaches to disease prevention for their populations and provided social support to families and individuals in need.^52^ Communities and civil society organisations also played vital roles in advocating for essential services for marginalised groups and individuals. New roles and relationships were forged within and beyond communities as they supported local responses to food, water, shelter, and safe transportation, among other needs.^3^ ^52^ ^53^

Women were at the forefront of many of these community led initiatives during the pandemic, even if their role was often not acknowledged or made visible.^3^ ^53^ For example, women’s rights organisations were critical to the continuity of gender based violence response and support services for women and girls—offering alternatives to suspended services and innovating or increasing their service provision.^54^ Supporting women’s organisations and initiatives that are grounded in community contexts is particularly important as they often have detailed knowledge of the specific needs of local women and families and of contextually appropriate interventions to meet them.

Community and civil society responses, often resourced by women in unrecognised ways, are not a substitute or add-on to government responses. They are key contributors that require support to ensure successful responses to health crises and their social effects. Given the extent of often female volunteer labour involved, failure to provide support further marginalises women who are already negatively affected by the pandemic. Not only does the level and diversity of income sources for community and civil society response need to be addressed,^51^ but the role of women and women’s organisations within these responses to covid-19 (and other crises) must be recognised and supported.^55^ Such support would both lay the basis for fostering quick and contextually adapted responses in times of crisis and challenge patriarchal notions around women’s leadership and their role in the community, a key structural element of gender inequality.

## Governments must take sustained action

In responding to covid-19, governments in many African countries rapidly identified and attempted to reduce its social impacts on women and girls. Some strategies such as the use of hotlines to support victims of gender based violence, emergency cash transfers for informal sector workers, and community based initiatives facilitating the provision of goods and services were implemented to meet women’s immediate needs. However, gender inequalities remain, and in some instances may have become worse, because efforts fell short of actual need, were biased, or failed to tackle the root causes of systematic gender imbalances. Gender inequality is both a personal and deeply structural form of social inequality that requires sustained action over time and across social systems to undo centuries of inequity.^56^

While it is imperative that we amplify the promising initiatives to address women’s immediate needs, more longer term planning and investments are needed to tackle the underlying norms and social and economic structures perpetuating gender inequality across Africa. The responses to covid-19 show that it is possible to advance gender equality, but more must be done to ensure it becomes the foundation for better preparedness and response to future health crises in the African region. Failure to act risks reversing the gains made by these initial responses.

**Key messages**

- Covid-19 exacerbated existing gender inequalities in Africa
- African leaders, in responding to the pandemic, pledged to prioritise gender equality and began to address the immediate needs of women and girls
- While these initiatives are important, meaningful investment in more sustainable strategies is needed to empower and protect women and girls
- Initial covid-19 responses related to preventing gender based violence, social protection, and community and civil society mobilisation can be built on to ensure sustained gains towards gender equality

## Notes

Contributors and sources: KB is a physician whose work focuses on promoting evidence informed decision making for a better access and a better quality of primary healthcare in Benin and other African countries. G is a gender and health systems researcher who co-led the gender and covid-19 research agenda setting process. MDJ is a social science researcher with experience working on gender mainstreaming in health policy and programmes. OA has over eight years of experience in research, evaluation, advisory work, and programme design in gender and international development. She was the coordinator for the thematic group on the social determinants for the gender and covid-19 collaboration, facilitating group discussions, and presenting the results in various forums. CF is a statistician and public health specialist with more than 20 years designing and implementing research on reproductive maternal neonatal and child health issues particularly in sub-Saharan Africa countries. TO has over 20 years’ experience providing specialist technical support to member states in the WHO African Region for the development, implementation, monitoring, evaluation, and promotion of equity oriented, rights based, and gender transformative approaches to strengthening health policies, plans and programmes. KM works for the vice-chancellor of the University of Global Health Equity in Rwanda and is interested in the intersection between public health and development economics. AB has served in high level government positions across the Rwanda health sector, including executive secretary of the National AIDS Control Commission, permanent secretary, and minister of health. She is on the African Union's Covid-19 Commission working group on gender and stigmatisation. KB and AG wrote the first draft, MDJ supported with background research, all authors contributed to conceptualisation, data curation, and reviewing and editing drafts. The authors alone are responsible for the views expressed in this article and they do not necessarily represent the views, decisions, or policies of the institutions with which they are affiliated.

## Notes

This article is part of a collection proposed by the United Nations University and the University of the Western Cape and commissioned by *The BMJ*. *The BMJ* peer reviewed, edited, and made the decision to publish these articles. Article handling fees are funded by Bill and Melinda Gates Foundation and UN University-International Institute for Global Health.

Publisher’s note: Published maps are provided without any warranty of any kind, either express or implied. BMJ remains neutral with regard to jurisdictional claims in published maps.
